# Future Responses to Managing Muslim Ethnic Minorities in China: Lessons Learned from Global Approaches to Improving Inter-Ethnic Relations

**DOI:** 10.1177/00207020221097991

**Published:** 2022-05-19

**Authors:** Reza Hasmath

**Affiliations:** 3158 University of Alberta, Edmonton, AB, Canada

**Keywords:** Ethnic minorities, securitization, prejudice reduction, socioeconomic inequalities, public policy, Xinjiang, China

## Abstract

Current policies to manage ethnic minority unrest in Xinjiang are not working, and do not address the core root causes behind ethnic tensions. Drawing upon lessons learned from global approaches to improving inter-ethnic relations, and factoring in China’s institutional behaviour and norms, this essay looks at policy responses that could be entertained by the state to improve the conditions of ethnic minorities in Xinjiang. It suggests that in the short-term (under a year) the state could be more responsible in using the big data it collects for targeted surveillance, in tandem with a community engagement approach. In the medium-term (one to three years), the state could employ practices to reduce ethnic prejudice by encouraging increased meaningful intergroup contact, and promoting a positive media portrayal of ethnic minorities. In the long-term (three years plus), improving the relative socioeconomic ethnic inequalities is paramount.

Scholarly evidence suggests that ethnic minority unrest in China has become a persistent and serious issue, especially amongst the Uyghur population—the largest group of the ten predominantly Muslim ethnic minorities—in the Xinjiang Uyghur Autonomous Region (XUAR).^1^ This unrest has periodically manifested in outbreaks of ethnic violence across China, from Beijing to the XUAR, since the early 2000s. The key question that China internally grapples with is, how should the state respond to the rise of ethnic minority unrest?

The Chinese state’s response^2^ has been a one-size-fits-all securitization strategy without appropriate institutional checks and balances. In Xinjiang, this can be seen in the state’s efforts to control the extent to which predominantly Muslim minority groups can engage in cultural and religious practices, the establishment of “re-education camps,” and a sophisticated mass surveillance system.^3^ Suffice it to say, maintaining large-scale security apparatuses in the XUAR is extremely expensive. Estimates suggest that, in 2017, the Chinese state spent 20 percent more on domestic security than on national defence, with RMB 57.95 billion (USD 9.10 billion) spent in Xinjiang alone.^4^

Foreign governments, global media outlets, and international human rights organizations have denounced the Chinese state’s heavy-handed approach towards the management of potential and actualized ethnic minority unrest.^5^ These external actors have largely politicalized the conditions of ethnic minorities in China within a civil and political human rights framework.^6^ Yet, a rights-based approach generally fails to problematize the situation in China’s domestic eyes. This is important since, ultimately, policies to manage ethnic minorities will be generated within China’s institutional and policy environment, and directed by Chinese actors. The Chinese government, led by the Communist Party of China, is more concerned about what its own citizens think—who have consistently provided a high degree of support to state actors in a host of key measures^7^—rather than the international community and applicable Western liberal critiques of China’s policies.

Nonetheless, the valuable insights and lessons learned from other nation states’ experiences managing ethnic minorities and improving inter-ethnic relations, are not being fully presented by international actors, and subsequently, not being factored by China in its own internal policy deliberation. This is particularly troubling since the Chinese state’s current approach is quite unlikely to reduce ethnic social unrest in Xinjiang; thus, episodes of ethnic tensions are likely to occur.^8^

In this essay, I will first discuss the contemporary context in which ethnic minority unrest has unfolded amongst the predominantly Muslim ethnic groups in China, with an emphasis on Uyghurs. I will thereafter outline policy proposals that could be entertained by the Chinese state in the short- (under a year), medium- (one to three years) and long-term (three years plus), factoring in China’s recent institutional behaviour and policy norms, and drawing upon lessons learned from global approaches to improving inter-ethnic relations.

## Context

The Xinjiang Uyghur Autonomous Region is situated in China’s northwest, occupying one sixth of the nation’s total land mass and holding one of the largest and most strategically important natural gas and oil reserves in Asia. The XUAR has a total population of approximately 24.87 million, of which the largest ethnic groups are the Uyghurs (∼46 percent), a Turkic, mostly Sunni-Muslim ethnic group; and the Han Chinese (∼39 percent), the national majority ethnic group.^9^ Relations between Uyghurs and Han Chinese in the region have been tense, with many historical and contemporary conflicts between them.

In fact, since 2009, Uyghur-linked violence has dramatically increased in the XUAR and in other parts of China. This was seen in July 2009 during the Ürümqi riots, which resulted in 197 deaths and 1,721 injuries. In November 2013, a car explosion by five Uyghurs in Beijing’s Tiananmen Square killed two and injured forty. This was followed by two separate outbursts of reported violence in Xinjiang between Uyghurs and Han Chinese on 26 and 28 June 2013, where thirty-five people were killed in total. In March 2014, a knife attack in Kunming Railway Station claimed the lives of twenty-nine individuals and injured 130. Chinese state media alleged that Uyghur militants were the assailants in both cases. In May 2014, two SUVs drove into shoppers, and bombs were thrown at them, in a Han-frequented Ürümqi market, killing forty-three and wounding more than ninety. On 18 September 2015, a knife attack in Aksu claimed the lives of nearly fifty individuals, and injured fifty. These are merely a few of the publicly reported acts of ethnic violence in the past decade, and they signify an increasing trend of ethnic tensions which has claimed the lives of hundreds, and injured thousands, across the XUAR and China.

### Root causes of Uyghur tensions in the XUAR

Among the main root cases of contemporary ethnic tensions in Xinjiang are policies that aim to temper Uyghur ethnic identity and Sinicize the group. This has involved limiting religious practices—often contra to Article 36 of China’s Constitution guaranteeing “religious freedom”—and reducing the importance of the Uyghur language in daily life. For instance, those under the age of eighteen years are not permitted to enter religious places such as mosques.^10^ Similarly, the state has prohibited all state employees from engaging in religious practices, which includes fasting during Ramadan, or wearing Islamic head scarves or coverings. The Koran can only be studied in specific government schools, and Imams are forbidden from teaching the Koran in private. Moreover, government informants frequently attend religious services, monitoring Imams and attendees.^11^ Additionally, Chinese authorities have gradually discontinued the use of the Uyghur language in most schools and universities, leaving Mandarin Chinese as the primary language of instruction. For many Uyghurs, these policies are viewed as efforts by the state to prevent them from accessing their sociocultural heritage.^12^

It is worth noting that the philosophical underpinnings of these policies can be traced back to the post-market reform era (1978 onwards), to notions of nationalism which became popular after the 1989 Tiananmen Incident and the 1991 collapse of the USSR. Chinese policymakers feared increased ethnic fracturing—which they viewed as a major contributor to the failure of the Soviet Union. Such fears came to pass with the 2009 Ürümqi efforts (as well as the 2008 Lhasa riots), and thus, Chinese policymakers sought to redouble their efforts to implement policies that actively increased the integration of different ethnic minority groups into a shared national, cultural identity—or, to paraphrase the words of Xi Jinping at the 19th National Congress of the Communist Party of China, “to forge a strong sense of collective consciousness for the Chinese nation.”^13^

Attention can also be drawn to the role that migratory patterns and socioeconomic inequalities have played in fomenting ethnic tensions in Xinjiang. Since the foundation of the XUAR, the Chinese state has resettled Han Chinese into the region; consequently, the Han population has increased from 6 percent in 1953 to 39 percent in present day.^14^ This population-transfer policy was informed by three objectives: (1) ensuring control of the region by diluting the proportion of restive ethnic minorities; (2) achieving economic development in the region; and, (3) easing population pressures in the eastern provinces.^15^ Following market reforms, migration has become more voluntary with Han Chinese being drawn to new employment and business opportunities in Xinjiang. The results of these migratory shifts have been a rapid urbanization of Xinjiang and the development of an ethnically segmented labour force.

Within the XUAR, Han Chinese have generally settled in wealthier urban regions in the north, whereas Uyghurs tend to comprise the majority of the population in rural areas and poorer urban areas in the south. Additionally, from 1991 to 2010, the Uyghurs’ share of the urban population declined markedly in most major cities; this is despite the fact that Uyghur birthrates are approximately four times greater than Han birth rates.

There is also a troubling division of labour, with Uyghurs predominantly working in the low-wage primary sector. Han Chinese continue to occupy the majority of jobs in the higher-paying secondary and tertiary sectors, where wages are more than twice that of the primary sector. Furthermore, Uyghurs have struggled to find success in the growing private sector, with Uyghurs being far less likely to be self-employed than their Han counterparts. These labour market inequalities are highly problematic, as Uyghurs use Han Chinese experiences as a measuring rod for their own socioeconomic wellbeing.^16^ As such, until these labour market inequalities are rectified, Uyghur ethno-cultural consciousness will be heightened, and Uyghur-Han Chinese conflict will continue to play a significant role in the history of Xinjiang.^17^

Compounding these issues, meaningful interactions between Han Chinese and Uyghur communities are severely limited. There is a general distrust between the groups, heightened by spatial divisions throughout the XUAR at large, and notably visible within its urban cores. This observation is further qualified by my own interviews with Han Chinese and Uyghurs for studies conducted over the past two decades. Seldom did I find that Han Chinese frequented “Uyghur areas” of Ürümqi, for example; and to be fair, this was often the case in the reverse. The lack of meaningful interactions between the groups can be understood as a function of wage and economic wealth, whereby the relatively lower-wage Uyghurs and higher-wage Han Chinese live in communities they can afford, thus creating spatial divisions that limit opportunities for meaningful daily contact.

For the majority of Han Chinese, in the absence of meaningful interactions with ethnic minorities, their perception of groups such as the Uyghurs is shaped by a commodified single story presented in mass media, often leading to the formation of negative stereotypes.^18^ There are countless examples of practices that present a packaged and commodified version of Uyghur ethnic identity, involving songs, dance, costumes, and food. These presentations can be seen at major events such as the annual Chinese New Year celebrations, or the 2008 Beijing Olympics, as well as in more everyday occurrences in ethnic minority restaurants or in official media sources. In contrast to the commodified portrayal of ethnic minorities, Han Chinese in comparable situations are portrayed wearing Western suits and dresses, symbolizing sophistication and modernity. Therefore, while ironically intended to improve inter-ethnic relations and showcase inter-ethnic harmony, public practices presenting the “ethnic minority” generally serve to draw mass attention to the differences between “backwards” and “exotic” minority cultures, and the “modern” and “advanced” Han Chinese culture. This issue is doubly problematic in the case of the Uyghurs whose cultural identity is intricately tied to a religious (Islamic) identity that has often been presented to the public as something dangerous that must be carefully managed.

## State reaction to Uyghur unrest in the XUAR

The state has reacted to Uyghur unrest in the XUAR with oscillating soft and hard policy responses. The hard policy approach, as previously hinted, is primarily a sophisticated security apparatus. This includes collecting Uyghurs’ biometric data—e.g., DNA, fingerprints, and face scans—as well as creating a series of checkpoints where Uyghurs are required to produce their phones for inspection, and be checked for materials that may be deemed “inappropriate.”^19^ Those individuals flagged as potentially engaging in “religious or political transgressions,” as defined by the state, are sent to “re-education centres.” Given there are no stringent guidelines for assessing and determining potential “transgressions,” misinterpretations by local officials have led to high numbers of individuals being sent to “re-education centres.” Also, a grid-style social management system has been rolled out, with communities in Xinjiang being divided into zones. Subsequently, a group of party members—with a high latitude of discretion to implement local tactics and strategies—is assigned to each zone, where they are responsible for monitoring and conducting surveillance on activities that are deemed threatening to “social stability.”

Fundamentally, the soft policy response is a strategy of community support and investment, which in the XUAR context is characterized by funding the building and maintenance of mosques. According to the State Information Office, there are over 20,000 mosques in Xinjiang, which makes this endeavour relatively significant. Research in Indonesia, a state with a large Islamic majority, suggests that investment in local religious institutions can reduce the effects of inter-ethnic socioeconomic inequalities, and reduce the odds of local violence.^20^ Religious institutions decrease violence resulting from local grievances in two ways: First, they provide local public goods. Second, they act as an “information bridge” between the local population and the government, allowing for non-violent management of potential discontent. One final aspect of the soft policy approach is the state providing preferential policies for ethnic minorities in a slew of social and welfare provisions^21^—most notably in education, where provisions consist of bursaries, scholarships, and lower exam score requirements for university admission.

In the present day, the state has employed a hard policy approach to suppressing ethnic tensions in the XUAR, although a soft policy approach will inevitably return in the future. What is troubling is that neither hard nor soft policy approaches employed by the Chinese state have meaningfully addressed the root causes of tensions amongst the Uyghurs. Future policies should be aimed at effectively addressing the inherent sources of ethnic tensions in the XUAR. Below, I outline these potential responses within short-, medium-, and long-term strategies.

## Short-term strategies (under a year)

Short-term policy changes are inherently limited in addressing serious systemic issues such as Uyghur socioeconomic inequalities relative to Han Chinese, or the commodification of their ethnic identity. Nevertheless, securitization strategies can be improved to reduce present and future ethnic minority resentment, and hostility, in the XUAR. Drawing upon lessons learned and best practices from other jurisdictions, China is in a position to refine its mass data surveillance approaches by precisely targeting at-risk individuals susceptible to radicalization, and to embrace a community engagement plan. The adoption of these strategies, in sum, could yield improved success in reducing ethnic tensions.

Nations such as the UK, Canada, USA, Japan, and South Korea have increasingly used big data to analyze the potential for individual radicalization. The reality of the situation is that big data analytics represents the “greatest opportunity to increase the effective delivery of counter-terrorism,” by pinpointing “radical roots” using tools to “identify informal networks, emerging topics, influencers, links between individuals, groups, or concepts and sentiment analysis.”^22^ For instance, research conducted into lone-wolf radicalism has shown the importance of counter-terrorism services being attuned to discrete signals that an individual or group with radical intent will almost always give off.^23^ From normative and ethical perspectives, one might justifiably be inclined to hold deep reservations about the use of these surveillance methods, especially considering the inherent human and machine learning biases that shape big data collection and analysis.

Considering the value of these tools, and that the Chinese state already possesses one of the most sophisticated surveillance systems, it would be quixotic to assume that the state will simply cease to continue these practices. Notwithstanding, this surveillance apparatus should not be used to aid in a mass, one-size-fits-all crackdown on core elements of Uyghur identity, Islam, and the Uyghur language—an approach the state has adopted with the appointment of Chen Quanguo as Party Secretary of XUAR in 2016.^24^ The state’s current surveillance approach has created significant resentment amongst the Uyghur population, even amongst those who have attempted to integrate into the People’s Republic of China, and it has increasingly heightened the group’s ethnic consciousness.^25^ This acute ethnic consciousness, combined with lower socioeconomic outcomes relative to Han Chinese, could lead to increased radicalization in the future. China’s counter-terrorism policies toward the Uyghurs, “since 2016 have taken on an exclusionary character that verges on characterizing them as *Homo Sacer.*”^26^ Instead, if a surveillance system must be used, it should be reserved for targeting specific individuals who can be credibly regarded as at-risk for radicalization and committing violence, with the appropriate institutional checks and balances—which, for the most part, has been absent in the current setup in the XUAR.

Another valued global practice for improving ethnic relations is a community engagement approach. Community engagement is a delicate process that involves government and security services forming connections with communities deemed at-risk for radicalization and violence.^27^ Interestingly enough, the Chinese state has generally not incorporated this strategy into their management of ethnic minorities in the XUAR.^28^ Whilst community outreach in the XUAR will be challenging since there is, understandably, significant distrust between the Uyghurs and Han Chinese (and by extension the Uyghurs and state institutions), it would be a mistake not to employ this approach.

There are two prominent advantages to utilizing community engagement strategies in the XUAR context. First, communities can potentially act as early warning systems for security services by supplying information or concerns they may have about certain individuals or small groups within the community. This information can be invaluable—notably when combined with surveillance data—for precisely discerning individuals or small groups who may be at-risk for radicalization.

Second, communities are well-positioned to address grievances—real and imagined—that enable radicals’ message to resonate within the community. Community leaders, in particular, can filter radical actions through a lens that de-emphasizes the message explicitly or implicitly. In the XUAR, this could include Imams and other community leaders spreading an anti-radicalization message, which emphasizes that the state is listening to, and actively addressing, grievances facing the community.^29^ This, of course, requires actual substantive efforts (and outcomes) by the state to address the grievances motivating Uyghurs’ unrest; otherwise, opinion leaders’ messages are likely to be dismissed as “cheap talk,” or “paying lip service” to state officials who do not represent their interests.

Recent evidence suggests that the state is already beginning to change its approach to managing Muslim ethnic minorities in Xinjiang, including purportedly ending the policy of “re-education centres.”^30^ While this is a positive development, the post-2016 logic of treating all Uyghurs as a potential threat is still evident in the security architecture in the XUAR. Consequently, all of the aforementioned negatives associated with this approach are still in effect. It is paramount that the state change its policy philosophy in this regard, and adopt a more responsible usage of big data and targeted surveillance technologies, in tandem with a community engagement approach that sets the tone for increasing social trust between ethnic minority groups and the Han Chinese majority in the medium- and long-term.

## Medium-term strategies (one to three years)

Intergroup contact has become largely accepted in the social sciences literature as an essential component for reducing prejudice, increasing social trust between ethnic groups, improving between-group relations, engendering forgiveness for past transgressions,^31^ and creating a more differentiated perception of the outgroup. Thomas Pettigrew et al. found that the optimal conditions for intergroup contact—equal status, common goals, no intergroup competition, and authority sanction—facilitate prejudice reduction.^32^ Cross-group friendships are particularly effective at promoting strong and enduring positive attitudes towards the outgroup.^33^ These effects are usually generalized beyond the confines of an individual’s relationship with members of another group, to the outgroup at large. Additionally, these positive effects can occur, although to a lesser extent, merely by having an ingroup friend with outgroup friends.

In the XUAR context, Han Chinese and Uyghurs reside in relatively segregated communities—largely attributed to a function of the differential wage and economic wealth outcomes between the groups, as discussed earlier—and rarely interact in a meaningful way. Furthermore, even when the state has implemented policies expressly designed to create environments where positive inter-ethnic interactions can occur, they seldom have this effect. Timothy Grose found that in the “Xinjiang class”—a program that funded Uyghurs from Xinjiang to attend secondary school in China’s wealthier eastern cities—meaningful interaction between the groups, despite being officially encouraged, rarely materialized.^34^ While the reasons for this vary, one of the most troubling is that, according to Yangbin Chen, “school authorities even set rules to officially discourage interaction, ostensibly in order to avoid potential ethnic conflicts.”^35^ As a consequence, Grose’s Uyghur informants reported often having uncomfortable interactions with Han students and teachers. Lin Yi encountered a similar environment in an ethnically merged school in Ürümqi: Han Chinese students and staff frequently discriminated against Uyghur students, believing them to be “inferior,” “uncivilized,” “barbaric,” and “unhygienic.”^36^ Even though some of the Uyghur students Yi interviewed reported wanting to have Han Chinese friends, they felt this was impossible due to the Han Chinese fearing them. This was similar in the context of elite universities in China.^37^ These outcomes are unsurprising, as it is probable that Han Chinese students, lacking prior meaningful interactions with Uyghurs, have primarily developed their perception of Uyghurs through the internalization of stereotypes and prejudices evoked by mass media or Han Chinese adults they interacted with at a younger age (e.g., family members or teachers).

The absence of meaningful interaction, and the extent to which existing prejudices taint inter-ethnic relations, does not bode well for reducing inter-ethnic tensions in the XUAR. It must be reiterated that in the absence of meaningful interaction, the two groups primarily learn about the other through media representations.^38^ As noted, ethno-cultural festivals and the mass media commonly depict Uyghurs in a highly commodified fashion, and sometimes, in the case of mass media, as a dangerous threat.^39^ Addressing these issues from a policy standpoint is a difficult proposition, especially considering the existing tense ethnic landscape in the XUAR. Nevertheless, there are potential strategies that the Chinese state could implement to increase intergroup contact and reduce ethnic prejudice.

Recent research in contact theory has suggested that, in much the same way as negative media portrayals of outgroup members can increase bias towards that group, positive portrayals can reduce prejudice.^40^ Although vicarious contact in this form has fewer positive consequences, it can still help to reduce prejudice and improve sentiments towards the outgroup. Significantly, this has been found to occur even in scenarios which involved ethno-religious conflicts such as between Israelis and Palestinians, or between ethno-racial groups in Africa.^41^ An additional advantage of vicarious contact via mass media is that it can influence a significant number of viewers, allowing the positive effects to occur faster at a population-wide level.^42^ Thus, the state should implement policies to change how Uyghurs are represented in the heavily state-controlled mass media.

First, media and ethnocultural festival portrayals of Uyghurs in a highly commodified manner should be significantly reduced, and in the best-case scenario, eliminated. The state should discourage negative media portrayals of Uyghurs. For example, existing acting roles for Uyghurs tend to erroneously depict members of the group in an “Orientalist” fashion, as “exotic” or “warlords” from Western regions or Islamic nations. The state can encourage positive media portrayals of Uyghurs by ideally depicting friendships between Uyghur and Han Chinese characters. This, in part, could be achieved in a hyper fashion through direct, or indirect, state financial support for media—television, books, films, or video games—that engages in this type of positive representation. It could also be achieved through direct state directives to media outlets—which is commonplace practice in China—instructing them to dampen down negative portrayals of Uyghurs and their cultural and/or religious practices. Vicarious contact and ending negative portrayals of Uyghurs are important initial steps in reducing prejudice between the groups; however, they will likely be insufficient unless paired with meaningful between-group contact.

Despite the failures that have occurred to date, schools will likely continue to be the best environment to foster direct contact between ethnic minorities and Han Chinese. This is largely because ethnic stereotypes and prejudicial attitudes are deeply ingrained by adulthood, reducing the extent to which even the best interventions can mitigate them. Reforms, however, are critical. Teachers and staff will need additional training to equip them with the requisite skills to properly teach Uyghur–Han Chinese mixed classes. Furthermore, extracurricular activities such as mixed ethnic sports teams can be beneficial in generating positive between-group feelings.^43^ Pilot programs will likely be necessary to determine the optimal strategy for these schools, with issues such as language(s) of instruction, and curriculum taking priority.

Most of these reforms can be instituted within the next one to three years. However, many of the effects of these policies will not be felt until significantly into the future. Hence, the state will need to adopt a long-term view of inter-ethnic integration, in addition to monitoring the progress of their initiatives.

## Long-term strategies (three years plus)

Relative socioeconomic inequality arguably plays the biggest role in fomenting ethnic minority unrest in the XUAR. Although Uyghur earnings have increased in the post-market reform era, they have not comparatively kept pace with those of the Han Chinese, generating feelings of resentment.^44^ As discussed, Uyghurs have predominantly found employment in the far lower-paying primary sector, and have been unable to make inroads into the higher-paying secondary and tertiary sectors. The inclination may be to presume that this is a result of differences in Uyghur and Han Chinese formal human capital. Yet, even when Uyghurs have equivalent or greater levels of formal education, robust statistical analysis suggests they continue to struggle to find high-paying and high-status employment; moreover, when Uyghurs do manage to find employment in higher-paying sectors, they continue to receive lower renumeration than their Han counterparts.^45^ Multiple explanations for ethnic job segregation and the ethnic earnings penalty have been discussed in the academic literature. Wenfang Tang et al. contend that Uyghurs’ lower levels of Mandarin attainment and/or poorer Mandarin skills, in part, explain why Uyghurs are unable to translate their formal human capital into labour market success.^46^ Exclusionary discrimination against Uyghurs based on Han Chinese employers’ prejudice regarding work ethics, culture, and religion also plays a significant role.^47^ Furthermore, both Han Chinese and Uyghurs rely on network ties to find job information, and the weaker quality of network ties among Uyghurs has a negative effect on their labour market outcomes.^48^ Lastly, employers may devalue ethnic minorities’ hard skills on the basis that they receive preferential treatment in education, incorrectly signalling to employers a lower quality of formal qualifications and work productivity in contrast to Han Chinese.

Chinese authorities are cognizant of the role that socioeconomic inequalities play in ethnic minority development—although there is a risk of this changing at present^49^—and subsequently, the rise of ethnic tensions. This is part and parcel of the Chinese state’s explicit philosophical emphasis on promoting social and economic human rights above civil and political human rights. In this vein, historically, economic incentives have been one of the primary means employed to manage the Uyghur population. The underlying principle behind the authorities’ profound belief in this approach is that Uyghurs mainly desire a satisfactory economic life for themselves and their offspring. This is a reasonable assumption, but nevertheless, many of the state’s prior economic interventions, while improving Uyghur earnings, have in fact exacerbated the relative socioeconomic divide between the Uyghurs and Han Chinese.^50^ The primary beneficiary of prior state initiatives to develop the XUAR has disproportionately been the Han Chinese population, with Uyghurs receiving far fewer economic benefits. Han Chinese migrants have also been drawn to the region seeking to capitalize on new employment and business opportunities. Additionally, the cost of living in urban centres has increased, pricing many Uyghurs out of desirable locations, and helping to contribute to the spatial segregation that we witness today.^51^

Changes in the state’s approach to financial development in Xinjiang are therefore necessary. Amy Liu and Kevin Peters have suggested that one potential solution is to actively control the migration of Han Chinese to the XUAR.^52^ While this constitutes a somewhat heavy-handed solution, it may prove fruitful. Another potential solution is to shrink the size of the Xinjiang Production and Construction Corps, an administrative organ that draws significant Han Chinese migration, and primarily employ them. If Tang et al.^53^ are correct, improving Uyghurs’ Mandarin skills could be highly beneficial for improved labour market outcomes, but could also be an avenue fraught with challenges. State policies previously enacted under the guise of improving Uyghurs’ job skills and teaching the cohort Mandarin have often stifled their native language and ethnocultural practices. Thus, there is likely to be significant skepticism amongst the Uyghur population (from both younger and older age cohorts) towards new language policies. Unless handled properly, this may generate further resentment amongst the population.^54^

Addressing Han Chinese prejudice through policy instruments is a difficult proposition, but there are potential solutions, particularly in the state sector. Xiaowei Zang found that in state agencies with a redistributive organizational culture—such as trade unions, women’s federations, and youth organizations—Uyghurs benefit from an ethnic premium, as these organizations generally aim to demonstrate compliance with social equity principles.^55^ In contrast, state firms with a market-oriented culture tend to discriminate against Uyghurs when hiring. Zang suggests that this is the result of perceptions amongst Han Chinese employers about the poor productivity levels of Uyghur employees. Thus, the more market-oriented the state firm, the less likely they are to hire Uyghurs. One potential solution to this is the implementation of diversity quota requirements in state-run firms. This approach is not without its drawbacks, however. The Han Chinese majority already resents existing affirmative action policies that ethnic minorities receive. Additionally, should minority employees who are genuinely unqualified be hired, their potential failures could feed into existing prejudices which will be attributed to the entire ethnic group. Nevertheless, improving the treatment of Uyghurs in the state sector is only a partial solution, owing to the growing prominence of the private sector.

Private sector interventions are even more difficult to implement, and many of the initiatives designed to promote diversity in firms located in Western jurisdictions have not achieved meaningful results due to organizational inertia.^56^ Potential solutions could include tax incentives for private enterprises in the XUAR to hire ethnic minority candidates; in conjunction with curbing Han Chinese migration (and thus reducing the availability of Han Chinese labour), this could encourage, or even necessitate, firms’ hiring of Uyghur candidates. Policies designed to increase the number of Uyghur-owned business are also worth considering. New policies could include creating specific government loan and subsidy programs for Uyghur-owned businesses, reducing the monetary cost associated with receiving necessary certificates and documents, and providing greater public support for Uyghur businesses. Importantly, Uyghurs must be made aware of these policies, as there appears to be a knowledge gap between Uyghur and Han Chinese entrepreneurs—a reality that is also seen in Western jurisdictions.^57^

Improving the socioeconomic conditions of Uyghurs is essential for reducing ethnic tensions, and consequently, for limiting the potential for ethnic violence. From a generational standpoint, this will require greater social trust between Uyghurs and Han Chinese, and the development of robust intergroup social interactions and networks. However, in the relatively near-term, policies that directly improve Uyghurs’ socioeconomic lives should be implemented. This will not only help to alleviate ethnic minority tensions, it will signal to the Uyghur population that the Chinese state is committed to improving their quality of life from an economic and social standpoint. That is, the state will adhere to its foundational principles pertaining to social and economic human rights.

## Conclusion

Perhaps the most significant changes that Chinese authorities can make in their management of Muslim ethnic minorities is how they conceptualize the issue itself. Instead of perceiving Uyghur identity as a threat necessitating extensive control, the state should target the main root causes of Uyghur unrest. If long-term inter-ethnic harmony is to be achieved, oscillating between the current slate of soft and hard policy approaches must be reconsidered. These approaches, as conceived, create a cyclical pattern that guarantees ethnic tensions will continue to shape the development of the XUAR in the twenty-first century.

Implementing new policies that alter the present-day securitization strategy, reducing inter-ethnic prejudice, and improving Uyghurs’ socioeconomic conditions are essential to eventually ending ethnic tensions in the region. While there exists no foolproof set of policy prescriptions that can ensure inter-ethnic harmony, it is important for the Chinese state to reduce the exorbitant cost of securitization, and to improve the long-term stability and economic development of the XUAR.

International actors who are concerned about the management of Muslim ethnic minorities in the XUAR can engage with the Chinese state—at the ministerial level and above—by offering their own experiences and lessons learned from integrating ethnic minorities in their own contexts. They can offer advice for potential strategies that were successful (and unsuccessful) in reducing ethnic unrest. To wit, Chinese senior officials are aware of the failures (and less so the successes) of ethnic minority integration in global jurisdictions, from ethnic fracturing in the former USSR, to Black Lives Matter movements in the USA.^58^ Optimistically, this suggests there is some common ground between China and other national jurisdictions to foster a meaningful state-to-state dialogue on the treatment of Uyghurs in the XUAR.^59^ Nevertheless, global actors should be aware that politicalizing the conditions of Muslim ethnic minorities within a civil and political rights-based framework will continue to fall on deaf ears and/or elicit a negative reaction within China,^60^ particularly in the current environment of increasing nationalism under Xi Jinping. This is not necessarily to advocate that nation states should cease to promote the political philosophical ideals that they hold dear. Rather, it is a reminder that at the end of the day, China’s approach to managing ethnic minorities will be generated within the nation’s institutional and policy environment and its political philosophy of governance, and executed by Chinese actors.

## Notes

1. Xun Cao, Haiyan Duan, Chuyu Liu, James A. Piazza, and Yingjie Wei, “Digging the ‘ethnic violence in China’ database: The effects of inter-ethnic inequality and natural resources exploitation in Xinjiang,” *China Review* 18, no. 2 (2018): 121–154.

2. For instance, in leaked internal government documents, Chinese leader Xi Jinping has directed the administration to “show absolutely no mercy” in the XUAR, which he characterized as a “struggle against terrorism, infiltration and separatism.” See Paul Karp, “Australia’s foreign minister labels China’s treatment of Uighurs ‘disturbing,’” *The Guardian*, 17 November 2019, https://www.theguardian.com/world/2019/nov/18/australia-foreign-minister-labels-china-treatment-uighurs-disturbing (accessed 27 January 2022).

3. Sean R. Roberts, “The biopolitics of China’s ‘war on terror’ and the exclusion of the Uyghurs,” *Critical Asian Studies* 50, no. 2 (2018): 232–258.

4. *The Financial Times*, “Security spending ramped up in China’s restive Xinjiang region,” 12 March 2018, https://www.ft.com/content/aa4465aa-2349-11e8-ae48-60d3531b7d11 (accessed 18 October 2021).

5. Most prominently, members of the “Five Eyes” intelligence alliance, comprised of Australia, Canada, New Zealand, the UK, and the USA, have called for the end of arbitrary detentions and the mass internment of Uyghurs. In a similar vein, civil society organizations with an international reach, such as Human Rights Watch, Amnesty International, and the World Uyghur Congress or its affiliates (e.g. Uyghur American Association)—consisting of largely exiled Uyghurs—have re-energized their efforts to bring to light the dire human rights conditions for Uyghurs in China.

6. While a rights-based approach is important, using this framework in the People’s Republic of China has not been largely successful in affording transformative sociopolitical changes since the Communist Party of China came into power in 1949. To wit, China has consistently prioritized, in rhetoric and policy action, social and economic human rights over civil and political human rights championed by most Western states.

7. Sarah Eaton and Reza Hasmath, “Economic legitimation in a new era: Public attitudes about state ownership and market regulation,” *The China Quarterly* 246 (2021): 447–472.

8. Marie Tredaniel and Pak K. Lee, “Explaining the Chinese framing of the ‘terrorist’ violence in Xinjiang: Insights from securitization theory,” *Nationalities Papers* 46, no. 1 (2018): 177–195.

9. National Bureau of Statistics, *China Statistical Yearbook 2019* (Beijing: China Statistics Press, 2020). Note, another ∼12.5 percent of the population comprises the other nine predominantly Muslim ethnic groups (e.g., Kazakh, Hui, Kyrgyz, Tajik, and Uzbek).

10. Joanne Smith Finley, “The Wang Lixiong prophecy: ‘Palestinization’ in Xinjiang and the consequences of Chinese state securitization of religion,” *Central Asian Survey* 38, no. 1 (2019): 81–101.

11. S. Frederick Starr, ed., *Xinjiang: China’s Muslim Borderland* (Armonk, NY: M.E. Sharpe, 2004).

12. Rachel Harris, “Repression and quiet resistance in Xinjiang,” *Current History* 118, no. 810 (2019): 276–281.

13. Xinhua, “Full text of Xi Jinping’s report at 19th CPC National Congress,” 3 November 2017, http://www.xinhuanet.com/english/special/2017-11/03/c_136725942.htm (accessed 18 October 2021).

14. National Bureau of Statistics, *China Statistical Yearbook 2019*.

15. Nimrod Baranovitch, “The impact of environmental pollution on ethnic unrest in Xinjiang: A Uyghur perspective,” *Modern China* 45, no. 5 (2019): 504–536.

16. Xiaowei Zang, “Socio-economic attainment, cultural tastes and ethnic identity: Class subjectivities among Uyghurs in Urumchi,” *Ethnic and Racial Studies* 39, no. 12 (2016): 2169–2186.

17. Reza Hasmath, “What explains the rise of majority-minority tensions and conflict in Xinjiang?” *Central Asian Survey* 38, no. 1 (2019): 46–60.

18. Jia Jin, Guanxiong Pei, and Qingguo Ma, “They are what you hear in media reports: The racial stereotypes toward Uyghurs activated by media,” *Frontiers in Neuroscience* 11 (2017); Reza Hasmath, “The interactions of ethnic minorities in Beijing,” University of Oxford Centre on Migration, Policy and Society Working Paper 14–111 (2014): 1–26.

19. James Leibold, “Surveillance in China’s Xinjiang region: Ethnic sorting, coercion, and inducement,” *Journal of Contemporary China* 29, no. 121 (2020): 46–60.

20. Alexander De Juan, Jan H. Pierskalla, and Johannes Vullers, “The pacifying effects of local religious institutions: An analysis of communal violence in Indonesia,” *Political Research Quarterly* 68, no. 2 (2015): 211–224.

21. For further discussion, see Reza Hasmath and Andrew MacDonald, “Beyond special privileges: The discretionary treatment of ethnic minorities in China’s welfare system,” *Journal of Social Policy* 47, no. 2 (2018): 295–316; Bjorn Gustafsson, Reza Hasmath, and Sai Ding, eds., *Ethnicity and Inequality in China* (New York and Oxford: Routledge, 2021).

22. Andrew Staniforth, “Open source intelligence and the protection of national security,” in Babak Akhgar, P. Saskia Bayerl, and Fraser Sampson, eds., *Open Source Intelligence Investigation: From Strategy to Implementation* (Berlin: Springer, 2018), 17.

23. Edwin Bakker and Beatrice de Graaf, “Preventing lone wolf terrorism: Some CT approaches addressed,” *Perspectives on Terrorism* 5, no. 5/6 (2011): 43–50.

24. When XUAR’s Party Secretary Zhang Chunxian was pushed aside in 2016 over his failure to foster stability in the region, his replacement, Chen Quanguo, was given a relative carte blanche by the CCP to bring new forms of ethnic governance. Chen Quanguo brought many of the tactics he pioneered in the Tibet Autonomous Region to Xinjiang—what he called a “clenched fist” strategy to “stability maintenance work.”

25. *The Financial Times*, “China’s great game: New frontier, old foes,” 14 October 2015, https://www.ft.com/content/60f33cf8-6dae-11e5-8171-ba1968cf791a (accessed 18 October 2021).

26. Roberts, “The biopolitics of China’s ‘war on terror’ and the exclusion of the Uyghurs,” 251.

27. Rachel Briggs, “Community engagement for counterterrorism: Lessons from the United Kingdom,” *International Affairs* 86, no. 4 (2010): 971–981.

28. Adrian Cherney, “Police community engagement and outreach in a counterterrorism context,” *Journal of Policing, Intelligence and Counter-Terrorism* 13, no. 1 (2018): 60–79.

29. From the state’s perspective, there are limits to empowering local Imams and religious leaders. Generally speaking, this will be reserved exclusively to individuals who have a long track record of being “trusted,” and who understand the hidden rules of expected behaviour by the state. That is, such individuals will have a minimum acceptable level of agreeableness (from the perspective of the state) to state goals. See, for example, Jonathan Tam and Reza Hasmath, “Navigating uncertainty: The survival strategies of religious NGOs in China,” *Journal of Civil Society* 11, no. 3 (2015): 283–299.

30. *The Wall Street Journal*, “‘Admit your mistakes, repent’: China shifts campaign to control Xinjiang’s Muslims,” 6 February 2020, https://www.wsj.com/articles/china-shifts-to-new-phase-in-campaign-to-control-xinjiangs-muslims-11580985000 (accessed 18 October 2021).

31. Reza Hasmath, Benjamin Ho, and Solomon Kay-Reid, “State apologies and the rehumanization of social groups,” paper presented at Workshop on Ethics, Rights, Culture and the Humanization of Refugees (Edmonton, Canada), 7–8 February 2020.

32. Thomas Pettigrew, Linda R. Tropp, Ulrich Wagner, and Oliver Christ, “Recent advances in intergroup contact theory,” *International Journal of Intercultural Relations* 35, no. 3 (2011): 271–280.

33. Rhiannon N. Turner, Miles Hewstone, Alberto A, Stefania Paolini, and Oliver Christ, “Reducing prejudice via direct and extended cross-group friendship,” *European Review of Social Psychology* 18, no. 1 (2017): 212–255.

34. Timothy A. Grose, *Negotiating Inseparability in China: The Xinjiang Class and the Dynamics of Uyghur Identity* (Hong Kong: Hong Kong University Press, 2019).

35. Yangbin Chen, *Muslim Uyghur Students in a Chinese Boarding School: Social Recapitalization as a Response to Ethnic Integration* (Lanham, MD: Lexington Books, 2008), 116.

36. Lin Yi, “A failure in ‘designed citizenship’: A case study in a minority-Han merger school in Xinjiang Uyghur Autonomous Region,” *Japanese Journal of Political Science* 17, no. 1 (2016): 22–43.

37. Reza Hasmath, “The education of ethnic minorities in Beijing,” *Ethnic and Racial Studies* 34, no. 11 (2011): 1835–1854.

38. Jin et al., “They *are* what you hear in media reports.”

39. *The Independent*, “Islamophobia is on the Rise in China,” 10 April 2017, http://www.independent.co.uk/news/world/asia/islamophobia-china-rise-online-hate-speech-anti-muslim-islam-nangang-communist-party-government-a7676031.html (accessed 18 October 2021).

40. Loris Vezzali, Miles Hewstone, Dora Capozza, Dino Giovannini, and Ralf Wolfer, “Improving intergroup relations with extended and vicarious forms of indirect contact,” *European Review of Social Psychology* 25, no. 1 (2014): 314–389.

41. Charlotte Cole, Cairo Ali Arafat, Chava E. Tidhar, Wafa S. Zidan, “The educational impact of Rechov Sumsum/Shara’a Simsim: A Sesame Street television series to promote respect and understanding among children living in Israel, the West Bank, and Gaza,” *International Journal of Behavioral Development* 27, no. 5 (2003): 409–422; Rezarta Bilali and Johanna R. Vollhardt, “Priming effects of a reconciliation radio drama on historical perspective-taking in the aftermath of mass violence in Rwanda,” *Journal of Experimental Social Psychology* 49, no. 1 (2013): 144–151.

42. Vezzali et al., “Improving intergroup relations with extended and vicarious forms of indirect contact.”

43. Elena Makarova and Walter Herzog, “Sport as a means of immigrant youth integration: An empirical study of sports, intercultural relations, and immigrant youth integration in Switzerland,” *Sportwissenschaft* 44, no. 1 (2014): 1–9.

44. Hasmath, “What explains the rise of majority-minority tensions and conflict in Xinjiang?”

45. Sebastian Cherng, Reza Hasmath, and Benjamin Ho, “Holding up half the sky? Ethno-gender labour market outcomes in China,” *Journal of Contemporary China* 28, no. 117 (2019): 415–433; Andrew MacDonald and Reza Hasmath, “Outsider ethnic minorities and wage determination in China,” *International Labour Review* 158, no. 3 (2019): 489–508.

46. Wenfang Tang, Yue Hu, and Shuai Jin, “Affirmative inaction: Education, language proficiency, and socio-economic attainment among China’s Uyghur minority,” *Chinese Sociological Review* 48, no. 4 (2016): 346–366.

47. Reza Hasmath, “From job search to hiring to promotion: The labour market experiences of ethnic minorities in Beijing,” *International Labour Review* 150, no. 1/2 (2011): 189–201; Reza Hasmath and Benjamin Ho, “Job acquisition, retention and outcomes for ethnic minorities in urban China,” *Eurasian Geography and Economics* 56, no. 1 (2015): 24–43.

48. Xiaowei Zang, “Affirmative action, economic reforms, and Han-Uyghur variation in job attainment in the state sector in Urumchi,” *The China Quarterly* 202 (June 2010): 344–361.

49. *South China Morning Post*, “China’s ethnic groups face end to affirmative action in education, taxes,” 5 December 2019, https://www.scmp.com/news/china/politics/article/3040577/chinas-ethnic-groups-face-end-affirmative-action-education (accessed 18 October 2021).

50. Amy H. Liu and Kevin Peters, “The Hanification of Xinjiang, China: The economic effects of the Great Leap West,” *Studies in Ethnicity and Nationalism* 17, no. 2 (2017): 265–280.

51. Rune Steenberg and Alessandro Rippa, “Development for all? State schemes, security, and marginalization in Kashgar, Xinjiang,” *Critical Asian Studies* 51, no. 2 (2019): 274–295.

52. Liu and Peters, “The Hanification of Xinjiang, China,” 279.

53. Tang et al., “Affirmative inaction.”

54. See, for example, Hasmath, “What explains the rise of majority-minority tensions and conflict in Xinjiang?”

55. Zang, “Affirmative action, economic reforms, and Han-Uyghur variation in job attainment.”

56. Frank Dobbin and Alexandra Kalev, “Why diversity programs fail,” *Harvard Business Review*, July–August 2016.

57. Reza Hasmath, *The Ethnic Penalty: Immigration, Education and the Labour Market* (New York and Oxford: Routledge, 2012).

58. BBC, “US and China trade angry words at high-level Alaska talks,” 19 March 2021, https://www.bbc.com/news/world-us-canada-56452471 (accessed 18 October 2021).

59. See, for example, Reza Hasmath, *A Comparative Study of Minority Development in China and Canada* (New York and Basingstoke, UK: Palgrave Macmillan, 2010).

60. China’s response to global pressures has ranged from the old adage that other nations should respect, and refrain from directly interfering in, China’s domestic “internal” affairs, to comments by top-ranking officials characterized by the Standing Committee in Xinjiang calling legislation such as the Uyghur Human Rights Policy Act “a scrap of paper that will be swept into the garbage dump by the force of justice.” See Ministry of Foreign Affairs of the People’s Republic of China, “Foreign ministry spokesperson Hua Chunying’s remarks on the US House of Representatives passing the Uyghur Human Rights Policy Act of 2019,” https://www.fmprc.gov.cn/mfa_eng/xwfw_665399/s2510_665401/2535_665405/t1721334.shtml (accessed 27 January 2022); *The New York Times*, “China lashes out at U.S.’s action against mass incarcerations,” 18 June 2020, https://www.nytimes.com/2020/06/18/world/asia/china-trump-pompeo-xinjiang-uighurs-bolton.html (accessed 27 January 2022).

